# Environmental surveillance for *Salmonella* Typhi in rivers and wastewater from an informal sewage network in Blantyre, Malawi

**DOI:** 10.1371/journal.pntd.0012518

**Published:** 2024-09-27

**Authors:** Christopher B. Uzzell, Elizabeth Gray, Jonathan Rigby, Catherine M. Troman, Yohane Diness, Charity Mkwanda, Katalina Tonthola, Oscar Kanjerwa, Chifundo Salifu, Tonney Nyirenda, Chisomo Chilupsya, Chisomo Msefula, Nicola Elviss, Nicholas C. Grassly, Nicholas A. Feasey

**Affiliations:** 1 Medical Research Council Centre for Global Infectious Disease Analysis, Department of Infectious Disease Epidemiology, School of Public Health, Imperial College London, London, United Kingdom; 2 Department of Clinical Science, Liverpool School of Tropical Medicine, Liverpool, United Kingdom; 3 Malawi-Liverpool-Wellcome Programme, Kamuzu University of Health Sciences, Blantyre, Malawi; 4 Science Group, UK Health Security Agency, London, United Kingdom; 5 School of Medicine, University of St Andrews, St Andrews, United Kingdom; Institute of Tropical Medicine: Instituut voor Tropische Geneeskunde, BELGIUM

## Abstract

Environmental surveillance for *Salmonella* Typhi may provide information on the community-level dynamics of typhoid fever in resource poor regions experiencing high disease burden. Many knowledge gaps concerning the feasibility of ES remain, especially in areas lacking formal sewage systems. We implemented protocols for *S*. Typhi ES, including site selection and catchment population estimation, sample concentration and testing using qPCR for *S*. Typhi specific gene targets. Between May 2021 and May 2022, we collected grab samples and Moore swabs from 43 sites in Blantyre, Malawi. Catchment characteristics, water quality, and human faecal contamination (qPCR for Bacteroides HF183) were also recorded. Their association with *S*. Typhi detection was investigated using a logistic mixed-effects regression analysis. Prevalence of *S*. Typhi in ES samples was 2.1% (1.1–4.0%) and 3.9% (1.9–7.9%) for grab and Moore swab samples, respectively. HF183 was associated *S*. Typhi positivity, with a unit increase in log genome copies/microlitre increasing the odds of detection of *S*. Typhi by 1.56 (95% CI: 1.29–1.89) and 1.33 (1.10–1.61) in Moore swabs and grab samples, respectively. The location and timing of *S*. Typhi detection through ES was not associated with the incidence of typhoid fever reported in associated catchment populations. During this period of relatively low typhoid fever incidence, wastewater surveillance continued to detect *S*. Typhi in human sewage and wastewater suggesting that ES using natural river systems can be a sensitive indicator of transmission.

## Author summary

Typhoid fever is a major public health concern throughout many low- and middle-income countries, resulting in significant morbidity and mortality. In recent years, two typhoid-conjugate vaccines have been pre-qualified, with the World Health Organization recommending their targeted use in high incidence, endemic regions. However, quality assured information on the spatio-temporal distribution of typhoid fever is lacking, and scale-up of typhoid vaccination is progressing slowly. Environmental surveillance for *Salmonella* Typhi in sewage and wastewater may be a cost-effective and scalable approach to identify high-burden regions, thereby motivating vaccine introduction. However, many knowledge gaps regarding the use of environmental surveillance for *Salmonella* Typhi remain, particularly where human wastewater is unmanaged and its disposal reliant on informal drainage channels and natural river systems. Therefore, we conducted a study in Malawi, Blantyre to ascertain the feasibility of environmental surveillance throughout an urban, natural freshwater river system. We measured *S*. Typhi prevalence in wastewater contaminated rivers and ditches serving a mixed land use environment with diverse population densities. Whilst detection rates of *S*. Typhi were relatively low, and no obvious spatial pattern of distribution was observed, this study demonstrates that the presence of *S*. Typhi throughout the year. It suggests that wastewater testing for *S*. Typhi may offer a sensitive surveillance tool that complements clinical case reporting and may help identify areas to be prioritized for vaccination rollout.

## Introduction

*Salmonella enterica* subspecies *enterica* serovar Typhi (*Salmonella* Typhi) is an enteric bacterium responsible for typhoid fever, a globally important and life-threatening disease [1]. Typhoid fever is transmitted via the faeco-oral route, through direct (short cycle) or environmental (long cycle) transmission pathways typically associated with polluted water sources [2]. Symptoms include fever, nausea, headaches, and abdominal pain, and in severe cases can result in intestinal perforation [3]. Despite a reduction in the global incidence of typhoid fever in recent decades, *S*. Typhi remains responsible for an estimated >10 million annual cases of typhoid fever globally, causing approximately 117,000 deaths [4]. Typhoid fever is endemic in many low- and middle-income countries (LMICs) including sub-Saharan Africa [5] where access to safe water and effective sanitation infrastructure is inadequate [6].

The recent proliferation of antimicrobial resistant (AMR) and extensively drug-resistant (XDR) strains of *S*. Typhi [7], represent an emerging global public health concern and highlight the importance of effective and timely vaccine deployment throughout endemic regions [8]. Since 2018, the World Health Organisation (WHO) has prequalified three typhoid conjugate vaccines (TCV) and recommended their targeting at countries with the highest burden of typhoid disease or high burden of AMR *S*. Typhi [9]. Diagnosis of typhoid fever, however, requires diagnostic clinical microbiology capacity, through blood culture of suspected cases [10]. Blood culture is expensive, has imperfect sensitivity and is not widely available in typhoid endemic settings [11]. Therefore, the burden of typhoid fever is uncertain in many countries and often underestimated [12]. As such, there remains a paucity of reliable data with which to estimate the true burden of typhoid in low-income settings.

Alternative surveillance approaches may assist in identifying the spatiotemporal distribution of typhoid fever. Environmental surveillance (ES) of water and sewage contaminated with human faecal matter offers an anonymous, relatively low cost and sustainable approach to establish *S*. Typhi circulation in areas where blood culture capacity is limited [13]. As an aggregate sampling strategy, ES can survey thousands of individuals and has capacity to detect community wide transmission, including that of both symptomatic and asymptomatic cases [14]. Moreover, routine repeat sampling may provide valuable information on the temporal dynamics of typhoid and could provide an early warning of disease outbreaks [15]. A varied range of ES approaches have been successfully used on a suite of human disease causing pathogens including as part of the global polio eradication effort [16], and more recently for SARS-CoV-2 surveillance [17] and numerous enteric pathogens [18]. To date, most ES studies have sampled from formal piped drainage and sewerage systems or major wastewater treatment centres. However, high incidence, low-income countries often lack such networks, with human waste typically deposited into open drainage channels and local rivers. Therefore, the potential value of natural river systems as a method for typhoid surveillance and the sensitivity of methods to detect *S*. Typhi in these systems are unclear.

Blantyre, the second largest city in Malawi, is a mixed-use environment comprising areas of highly densely populated areas, local agricultural lands, and various natural mixed vegetation [19,20]. The city has a population of approximately one million people with the majority living in informal, unplanned settlements [21] and is served by the Queen Elizabeth Central Hospital (QECH) [22]. At present, formal wastewater drainage networks are limited and there are no operational wastewater/sewage treatment plants [23]. Therefore, the majority of the population rely on earthen or concrete pit latrines and septic tanks [21,24]. Moreover, river water is typically used for cleaning and cooking, and has recently been linked to environmental exposure to *S*. Typhi in Blantyre [25].

This study sought to assess the feasibility of utilising ES in an urban environment drained by a natural river system and investigate whether this approach might be a useful tool for confirming the presence and sustained transmission of *S*. Typhi within the local population. Secondly, we aimed to investigate whether it is possible for ES to contribute to the identification of local hotspots of clinical cases of typhoid fever. Additionally, we sought to explore the association between *S*. Typhi detection and ES catchment level environmental characteristics.

## Methods

### Ethics statement

This study was completed under ethics application P.10/19/2819, ethical waiver P.07/20/3089 from the University of Malawi College of Medicine Research Ethics Committee (COMREC), now part of Kamuzu University of Health Sciences. An additional waiver was provided by the Imperial College London Joint Research Compliance Office.

### ES site selection

**Fig 1:**
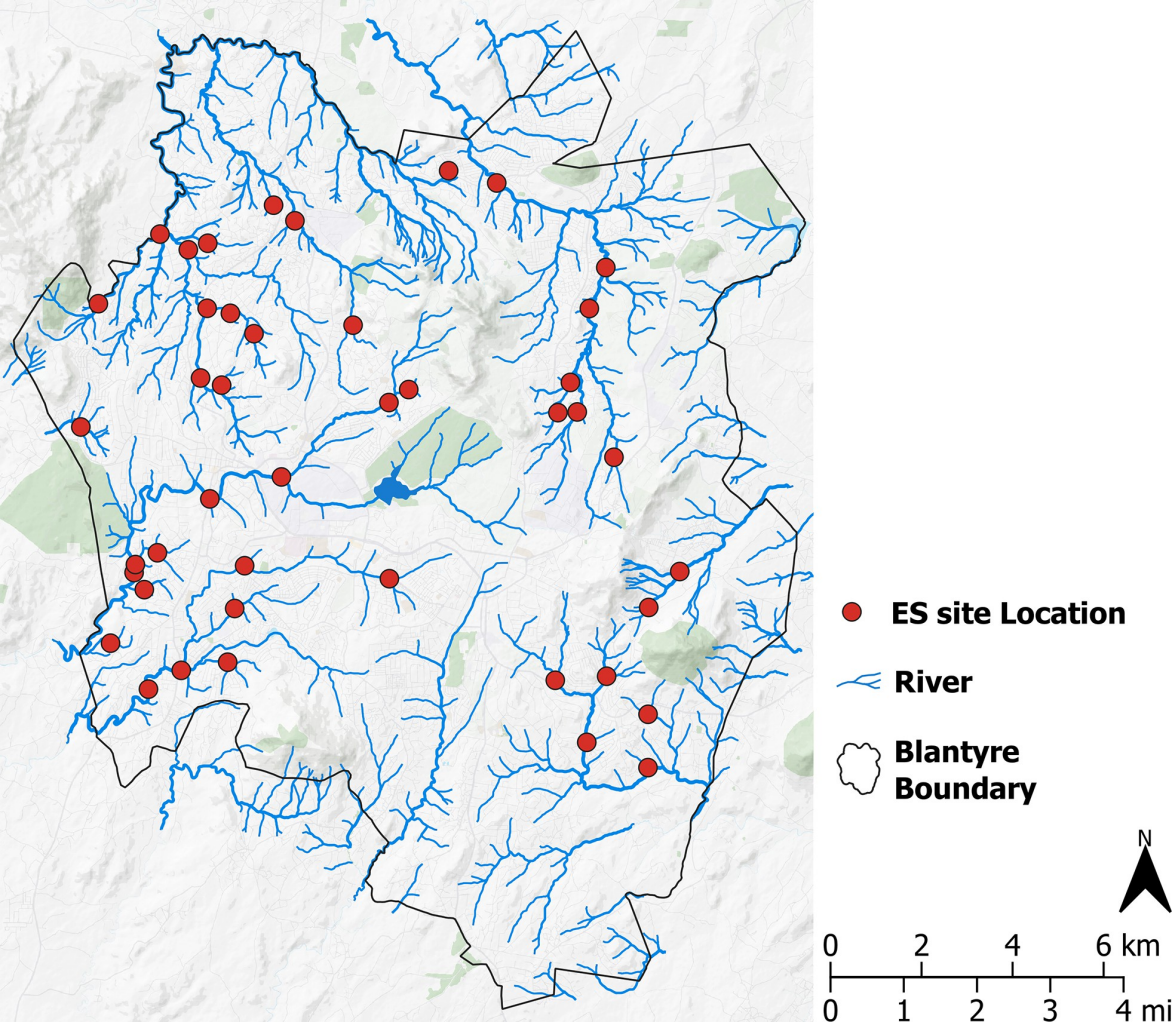
Spatial distribution of final 43 ES site locations. Map created in QGIS v3.22.4. Background map: CartoDB Positron layer accessed with QuickMapServices QGIS Plugin (https://carto.com/basemaps) made available under the Creative Commons Attribution (CC BY) 4.0 license.

Utilising a previously published standardised protocol using a remote, geographic information systems (GIS) based framework with geospatially referenced data, ES sites were systematically identified throughout Blantyre [26]. Briefly, OpenStreetMap river data (‘*blue-lines’*) were acquired and coupled with a high-resolution digital elevation model within ArcMap v10.7. Georeferenced hydrological surface layers were generated and river stream order was established using the Strahler stream order index classification approach [27]. Briefly, Stahler stream order is a categorical variable used as a surrogate for stream size. These data were used to establish a list of candidate ES sites, generate catchments and calculate population estimates based on the High-Resolution Settlement Layer (HRSL) dataset [28]. Using published power calculation estimates [26], and field-based inspections to investigate ES site suitability, candidate sites were screened and down-selected to a total of 43 ES sites (Fig 1). ES sites were selected based on the following constraints: broad geographic coverage, catchment population >1,200, sufficient river flow, safe accessibility, wide range of catchment sizes and anticipated year-round access based on local knowledge. Once selected, sites were stratified into 3 equal size classes (terciles) based on catchment population estimates and the proportion of various land uses calculated for each catchment.

### Sample collection and processing

**Fig 2:**
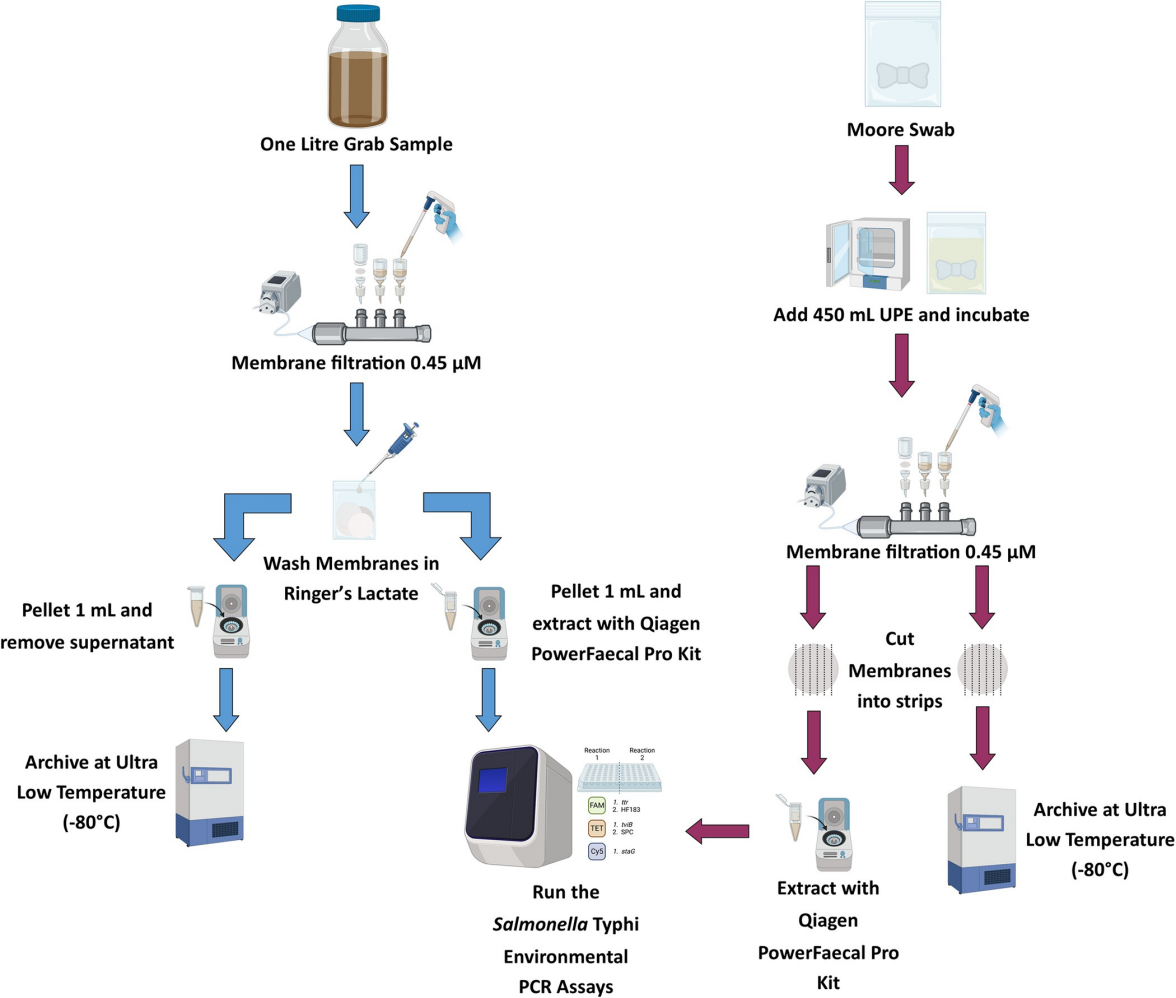
Flow diagram of sample processing after collection for both the water grab samples and Moore swabs from culture to PCR. Figure created with BioRender.com.

Sample collection and laboratory testing methods were based on protocols recommended by an expert advisory group established by the Bill and Melinda Gates Foundation following formal comparison of 8 different published protocols [29]. Repeat samples from 43 ES sites were collected each month between May 2021 and May 2022 (*i*.*e*. 13 months study period). Sample types collected consisted of one litre water grab samples, collected in autoclavable PPCO bottles, and composite sampling with the Moore swab, manufactured with sterile gauze and high-tensile braided fishing line [30]. Moore swabs were deployed 72 hours before collection each week, whilst grab sample collection was performed once per week at the same time as Moore swab collection, between 8 and 11am when possible. The full laboratory processing protocol is summarised in Fig 2. In short, grab samples were filtered on 0.45μM membranes which were eluted in Ringer’s lactate and spun down to produce pellets from which DNA was extracted using the Qiagen Powerfecal Pro kit (Qiagen). Moore swabs were incubated for 24 hours in Universal Pre-enrichment Broth (UPE), an aliquot of which was filtered on a 0.45μM membrane which went directly into extraction, again using the Qiagen Powerfecal Pro kit. For all DNA extraction batches, a no-template control (NTC) containing no sample was also included by extracting nuclease free water alongside samples.

The qPCR reaction used for these samples included two different reactions. The first included primers for ttr, tviB and staG to determine whether the sample was positive for *S*. Typhi. The second contained primers targeting HF183, a partial 16S rRNA gene sequence highly specific to human-associated Bacteroides dorei to act as a positive control for faecal contamination in a sample, and a commercially available Sample Processing Control (Eurogentec) to act as an extraction and inhibition control [31]. For all qPCR runs, the NTC was run in addition to a PCR negative, using the same nuclease free water as used to make the mastermix and primer/probe pool, and a positive control, using a genomically confirmed, anonymised clinical strain of *S*. Typhi [31] were also included. Full study protocols can be found at https://www.protocols.io/workspaces/typhoides.

### Typhoid fever hospital surveillance data

MLW has offered a quality assured diagnostic blood culture service to all febrile adult and paediatric medical patients presenting to QECH since 1998 [32]. In brief, blood is drawn under aseptic conditions and inoculated into a single aerobic bottle and incubated in an automated system (BacT/ALERT, BioMerieux, France). Blood culture bottles flagging as positive are processed using standard methods and Salmonellae identified by biochemistry and antisera using the Kauffmann and White scheme. Blood culture-confirmed typhoid fever cases reported to the QECH were recorded for the duration of the ES study. Moreover, additional cases identified through enhanced passive surveillance (Typhoid Vaccine Acceleration Consortium (TyVAC)) conducted during the ES were recorded and added for analysis.

### Statistical analysis

Sampling data was analysed for Moore swabs and grab samples separately by fitting univariate and multivariate mixed effects logistic regression models, using site-specific random effects to investigate associations between site and sample level characteristics (time of day collected, speed of flow, catchment land use, precipitation on the day of collection *etc*.) and both *S*. Typhi detection and the presence of human fecal matter. To identify spatiotemporal trends in detection and correlation with hospital reported cases of typhoid fever, we plotted monthly cases with monthly detection rates. Additionally, we assigned cases fractionally to catchments based on the overlaps of wards (known for cases) with catchments for comparison with binomial regression, using estimated incidence per million as a predictor for *S*. Typhi presence in a sample.

## Results

### ES sites and catchments summary

A total of 43 ES sites, stratified into 3 catchment population size classes, were identified. Estimated catchment area size and population estimates varied markedly, from 0.2 km^2^ to 33.8 km^2^ and between approx. 1,200 to 107,000, respectively (Table 1).

**Table 1:** Environmental Surveillance Site Characteristics.

Characteristic	Catchment Classification		
	Small	Medium	Large
Number of Sites	15	14	14
Median catchment area, km^2^ (range)	0.8 (0.2–1.6)	2.6 (1.1–7.3)	18 (1.9–33.8)
Median population (range)	5,961 (1,247–8,073)	17,451 (10,508–50,225)	80,935 (58,422–106,905)
Median population density, person/km^2^ (range)	6,720 (4,098–17,694)	6,232 (3,560–14,523)	4,941 (3,029–30,059)

Between May 2021 and May 2022, 533 grab samples and 594 Moore swabs were processed. Monthly sampling frequency varied significantly, between 4–23 (median: 13) and 3–48 (median: 14) for grab samples and Moore swabs, respectively. Approximately 96% of Moore swabs were successfully retrieved, however, retrieval rates varied significantly between ES sites (range 100%-60%).

### Spatial distribution of *S*. Typhi rates of detection

**Fig 3:**
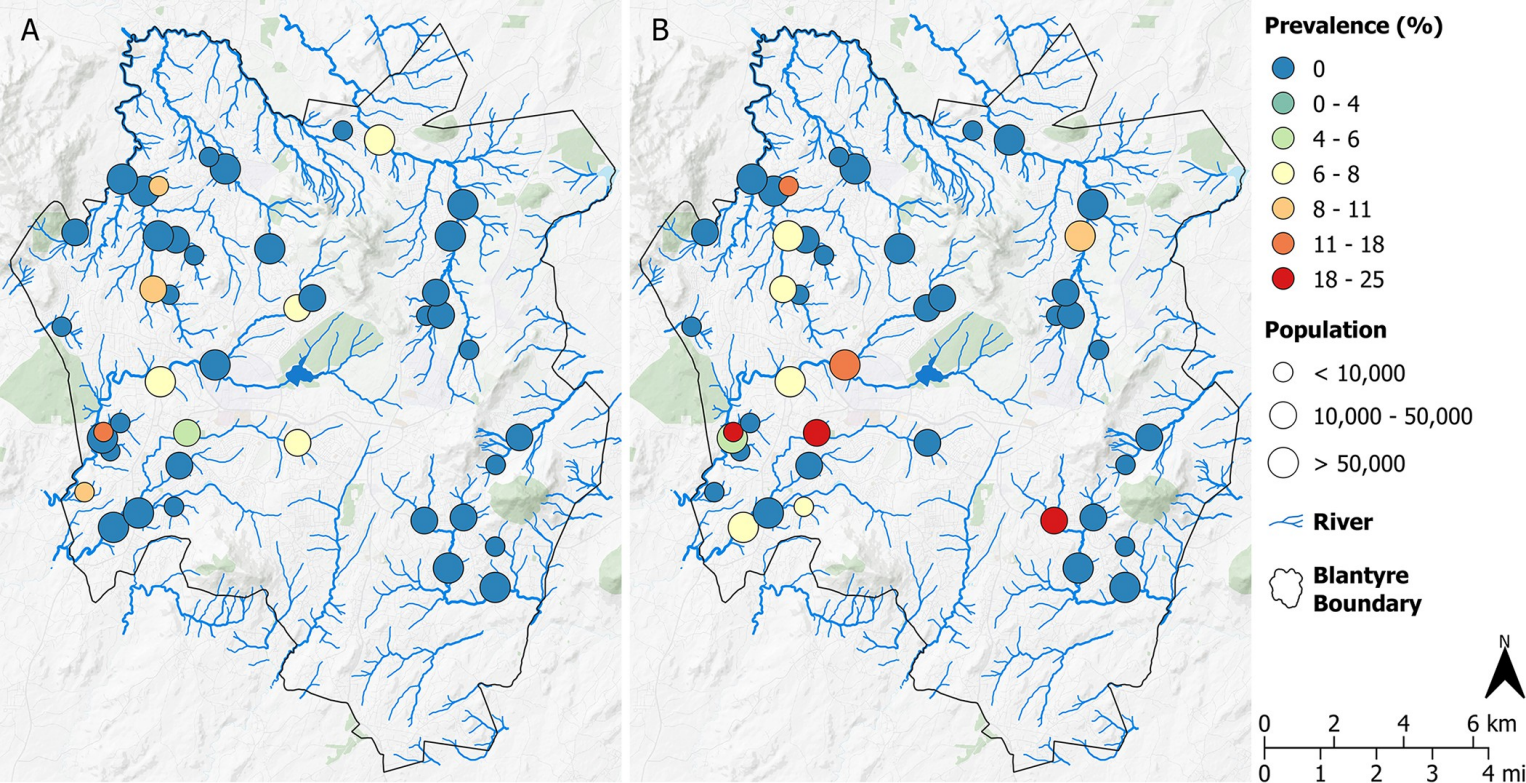
Geographic distribution of ES sites and prevalence (%) of *S*. Typhi detection for A) grab samples and B) Moore swabs in Blantyre. Circle radius proportional to the estimated catchment population. Maps were created in QGIS v3.22.4. Background map: CartoDB Positron layer accessed with QuickMapServices QGIS Plugin (https://carto.com/basemaps) made available under the Creative Commons Attribution (CC BY) 4.0 license.

A total of 11/533 (2.1%; 95% CI: 1.1–4.0) grab samples and 23/594 (3.9%; 95% CI: 1.9–7.9) Moore swabs tested positive for *S*. Typhi from 9 (20%) and 12 (27%) ES sites, respectively (Fig 3). The difference in prevalence was not statistically different (p = 0.158; S1 and S2 Tables). There appeared to be a higher rate of positivity in the south-west of the region (S1 Fig). All samples positive for *S*. Typhi came from 16 sampling sites. For grab samples, prevalence was noticeably lower from mid- and high-stream order ES sites (1.5%; 95% CI: 0.1–5.4 and 1.6%; 95% CI: 0.0–5.6, respectively) compared to low order streams (2.5%; 95% CI: 1.3–2.5). Similar results for Moore swabs were identified with highest rates of detection from low order streams (4.4%; 95% CI: 2.4–7.3) compared to mid- (2.3%; 95% CI: 0.0–6.7) and high-order steams (3.9%; 95% CI: 0.1–8.9).

### Faecal contamination

**Fig 4:**
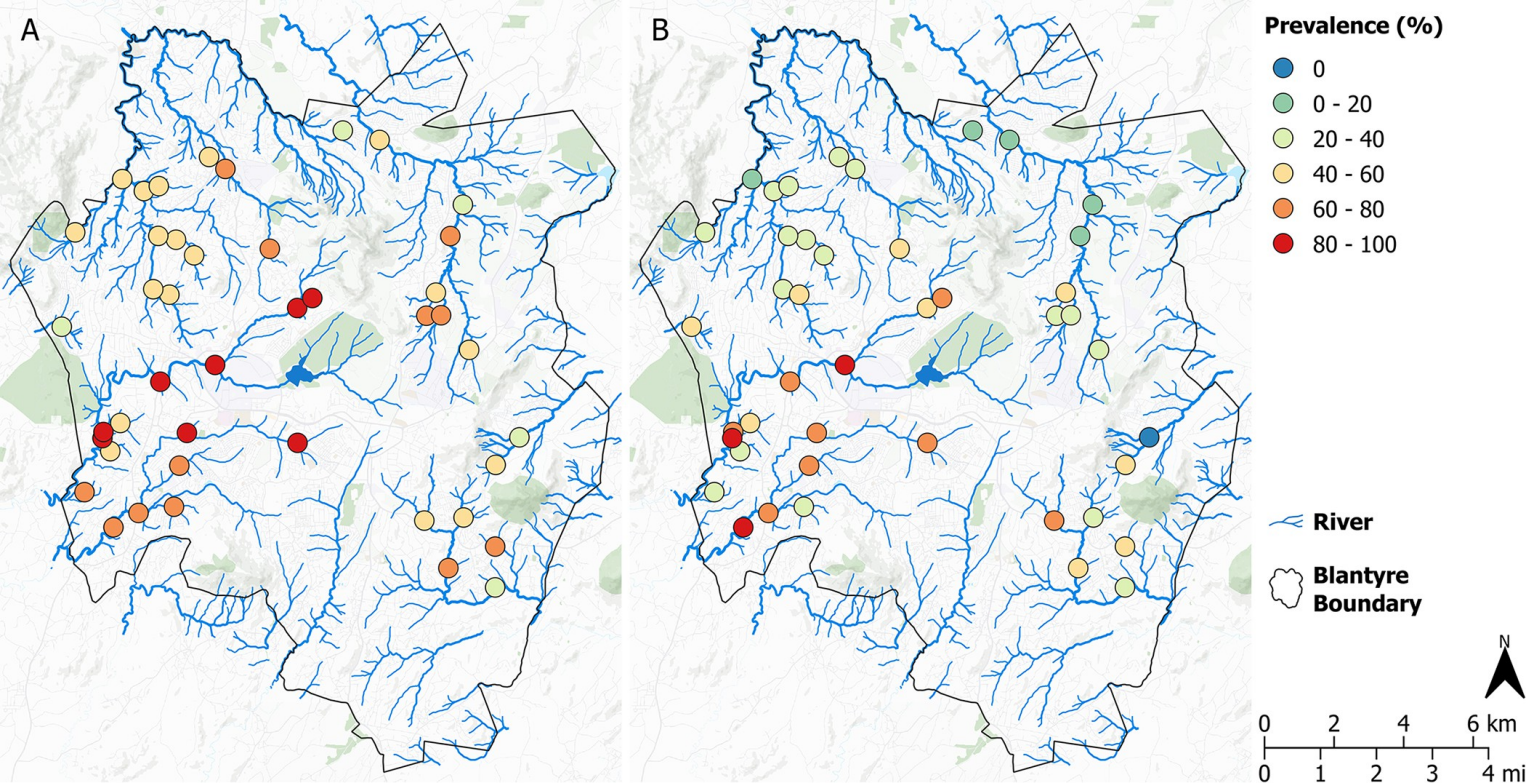
Geographic distribution of prevalence (%) of HF183 detection for A) grab samples and B) Moore swabs in Blantyre. Maps were created in QGIS v3.22.4. Background map: CartoDB Positron layer accessed with QuickMapServices QGIS Plugin (https://carto.com/basemaps) made available under the Creative Commons Attribution (CC BY) 4.0 license.

HF183 was detected at 43 (100%) and 42 (98%) ES sites for grab samples and Moore swabs, respectively (Fig 4). However, 45% of all samples were negative for HF183. No sites were positive for HF183 for all sample months; 13/43 (30.2%) of the sites were positive for HF183 <50% of the times they were sampled. Total precipitation on the day of sample collection was slightly negatively associated with HF183 detection, Grab sample OR 0.956 (95% CI 0.926–0.988), and Moore swab OR 0.962 (95% CI 0.929–0.996), consistent with dilution effects.

Presence of HF183 was significantly associated with *S*. Typhi positivity. Of the 277 HF183 positive Moore swab samples, 19 (6.9%) were also positive for *S*. Typhi, whilst of the 317 Moore swab samples negative for HF183, 4 (1.3%) were *S*. Typhi positive. Moreover, in univariate logistic regression, presence of HF183 increased the odds of *S*. Typhi detection by a factor of 4.61 (95% CI: 1.47–14.5). Where HF183 was present, an increase of one in the value of the natural log of HF183 genome copies increased the odds of detection by a factor of 1.56 (95% CI:1.29–1.89).

Similar results were seen for Grab samples, with 3.2% (11/342) HF183 positive samples also positive for *S*. Typhi compared with 0% (0/191) of HF183 negative samples. In univariate logistic regression, where HF183 was present, an increase of one in the value of the natural log of HF183 genome copies increased the odds of detection by a factor of 1.33 (95% CI:1.1–1.61).

### Variation with time and clinical disease incidence

**Fig 5:**
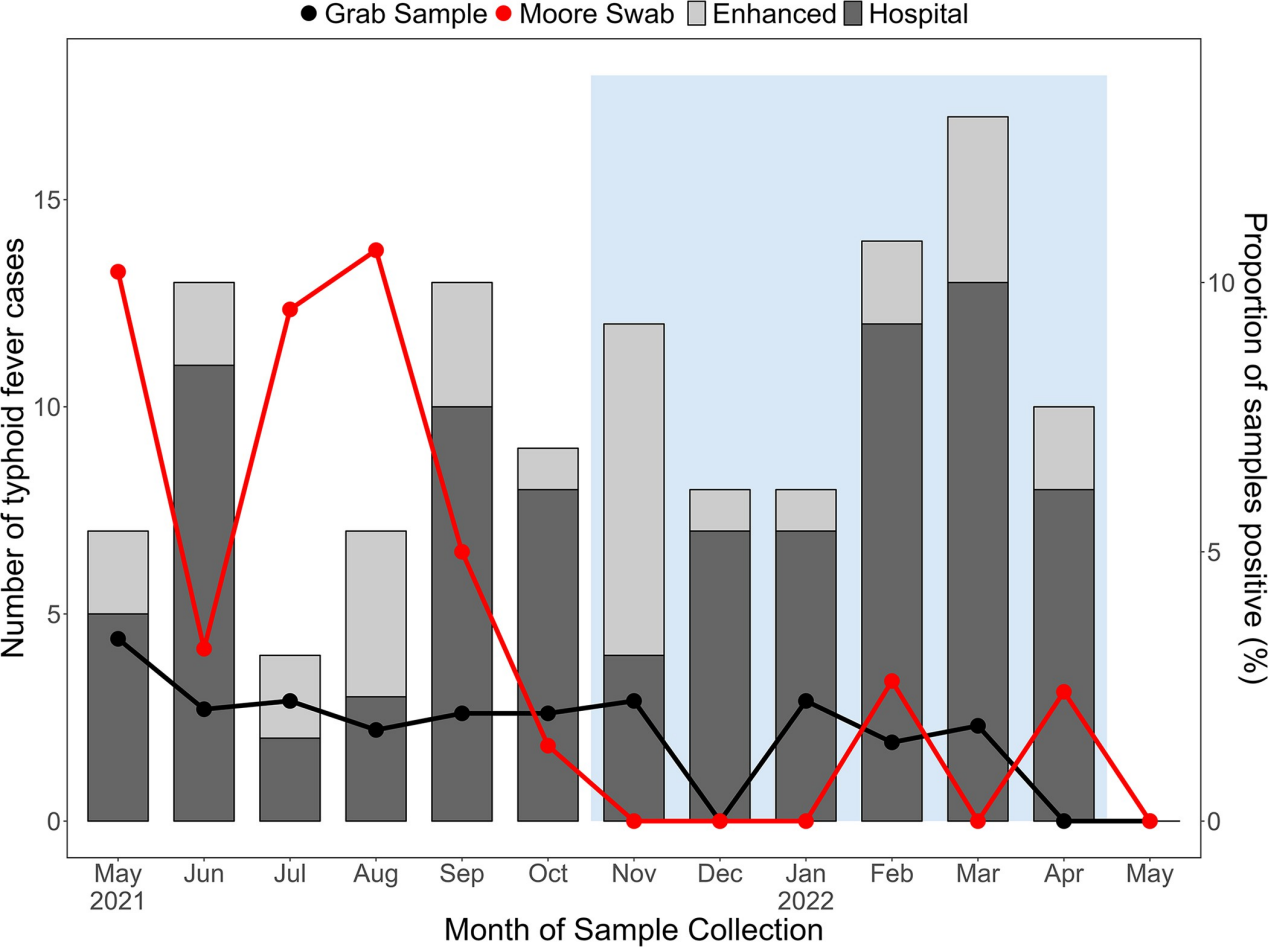
Monthly detection of *S*. Typhi in ES samples (right y axis) and the incidence of clinical cases reported (left Y axis) in Blantyre, Malawi. For the period May 2021 to April 2021 only, the monthly number of blood culture-confirmed typhoid fever cases reported among inpatient and outpatient resident in the study area (dark grey) with additional cases detected through enhanced passive surveillance at three primary health care centres shown in light grey. Light blue area indicates wet season period.

For Moore swabs, *S*. Typhi detection rates varied markedly throughout the 13-month study period with detection highest during May-October 2021 (Fig 5 and S9 Table). Little variation was recorded among grab samples with monthly rates <5% throughout. Overall, most positive samples (28/34; 82.4%) were recorded during the dry season. Monthly detection rates can be seen in Fig 5. No association between *S*. Typhi detection and clinical cases was observed (S9 Table).

**Fig 6:**
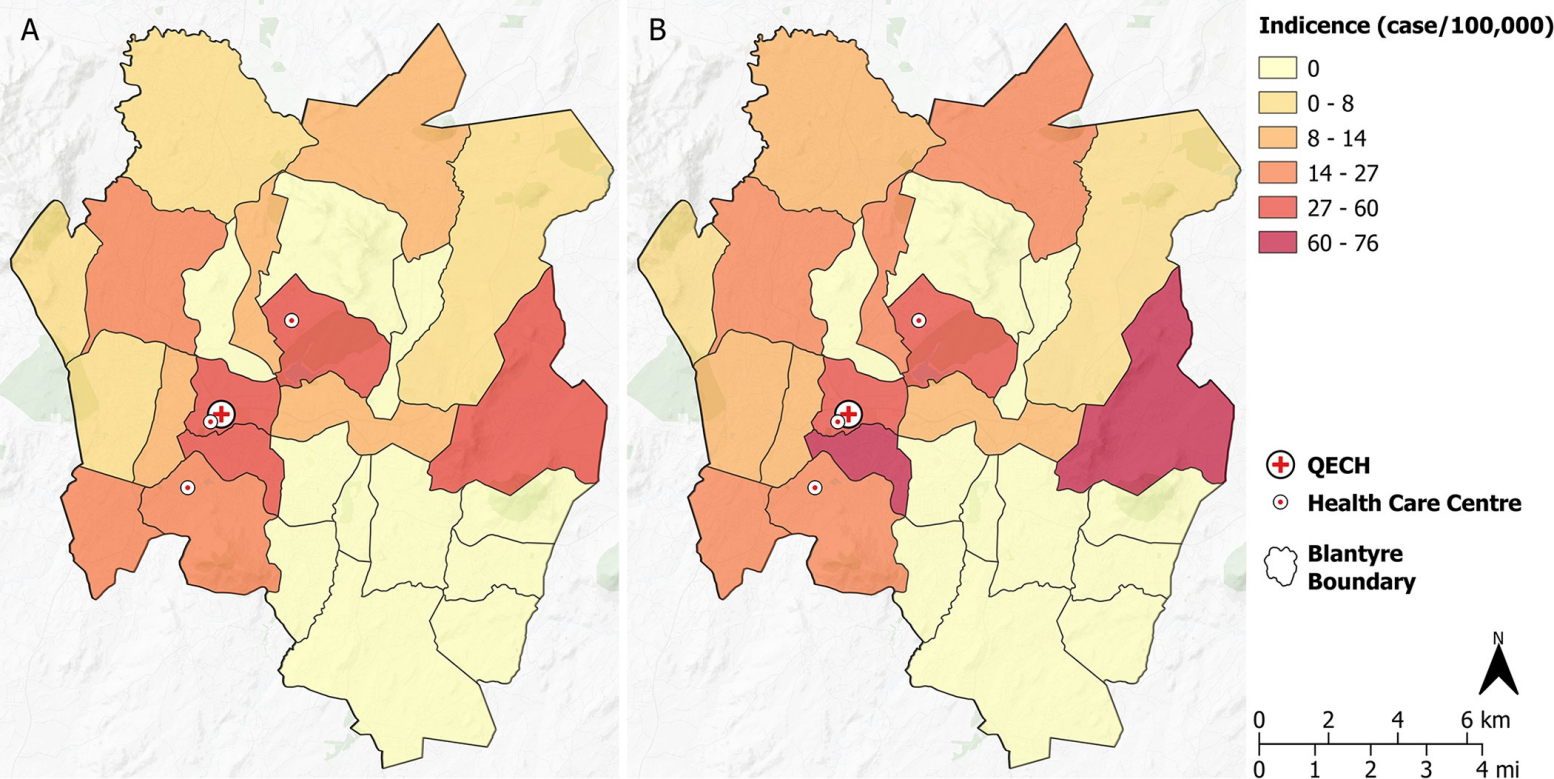
Geographical distribution of crude cumulative incidence rates of typhoid fever (cases per 100,000) in Blantyre from May 2021 to April 2022 using A) hospital reported cases, and B) additional cases through enhanced passive surveillance at 3 primary health care centres. The location of the QECH shown for reference. Maps were created in QGIS v3.22.4. Background map: CartoDB Positron layer accessed with QuickMapServices QGIS Plugin (https://carto.com/basemaps) made available under the Creative Commons Attribution (CC BY) 4.0 license.

Ward level crude incidence rates of typhoid fever is depicted in Fig 6. Fractions of cases are assigned to catchments according to the level of population overlap between each ward and catchment. This leads to an estimated number of cases per million population for each catchment, to be compared with the rate of positive samples among fecally contaminated samples (S2 Fig). No correlation between detections and estimated catchment incidence was identified for Moore swabs (OR:0.998; 95% CI: 0.993–1.00; p = 0.371) or grab samples (OR:0.999; 95% CI: 0.993–1.00; p = 0.697).

#### River sites vs sewage sites

Samples were predominantly taken from river sites, with just two sites classified as sewage network sites. *S*. Typhi detection was higher at these sites compared to river sites for both Moore swab and grab samples. For Moore swabs, 11 of 91 (12.1%; 95% CI: 7.07–23.3) sewage site samples were positive for *S*. Typhi, compared to 12 of 503 (2.39%; 95% CI: 1.27%-4.23) samples from river sites. For grab samples, 3 of 39 (7.69%; 95% CI: 1.75–22.5), and 8 of 494 (1.62%; 95% CI: 0.71–3.22) were *S*. Typhi positive for sewage and river sites, respectively. Site type was a significant predictor of *S*. Typhi, for both Moore swabs (OR 6.19; 95% CI: 1.27–30.2), and grab samples (OR 5.06; 95% CI: 1.29–19.9) when included in univariate mixed effects logistic regression. The significance of this predictor disappeared in a multivariate analysis where the log of the HF183 copy number was considered, consistent with the dependence being due to the greater quantities of faecal matter available at the sewage sites.

### Factors affecting *S*. Typhi detection

For Moore swabs, *S*. Typhi detection had a possible association with deeper river channels (OR:2.46, [0.996, 6.08], p = 0.051) and fast stream velocity (OR:2.82, [1.12–7.08], p = 0.031) in univariate logistic regression. These two effects disappeared in a multivariate analysis, in which the natural log of the HF183 genome copies was included. In addition, the proportion of ES catchment classified as institutional (OR:1.13, [1.00–1.28] p = 0.048) and utilities (OR:3.80, [1.39–10.40], p = 0.009) was also associated with Moore swab *S*. Typhi detection. Catchment population was not associated with *S*. Typhi detection (Grab samples: p = 0.598, Moore swab, p = 0.718), neither was the precipitation on the day of sampling (Grab samples: p = 0.480, Moore swab: p = 0.298). No trends were identified between physiochemical measurements and *S*. Typhi detection (S3 and S6 Tables).

### Factors affecting HF183 detection

For grab samples, HF183 detection was associated with increased stream velocity (OR:1.63, [1.10–2.40], p = 0.014). Moreover, of the physiochemical measurements, only the oxidation reduction potential had a small but significant correlation with HF183 positivity (OR:1.01, [1.00–1.01], p = 0.030). Several covariates related to catchment level land use area (high density permanent residences, commercial, utilities, institutional) had small but significant correlations with HF183 (S7 and S8 Tables). For Moore Swabs, larger proportion of commercial (OR:1.02, [1.01–1.04], p = 0.004), institutional (OR:1.01, [CI 1.01–1.02], p<0.0001) or utility-related (OR 1.09 [1.05–1.13], p = 8.1367e-06) land uses were significantly correlated with HF183. For both grab samples and Moore swabs, sewer sites were associated with HF183 detection compared to river sites (OR:2.80, [1.01–7.78], p = 0.049 and OR:5.73, [1.72–19.1], p = 0.004, respectively). Total precipitation was negatively associated with HF183 positivity, for Moore Swabs (OR:0.96, [0.92–0.99], p = 0.027) and grab samples (OR:0.95, [0.92–0.98], p = 0.007). Full univariate logistic regression analyses for association of various covariates with HF183 presence (with site specific random effects) can be found in S7 and S8 Tables. Temporal distribution of HF183 detection rates for both grab samples and Moore swabs are provided in S3 Fig.

## Discussion

We present results from a comprehensive, city-wide ES surveillance program for *Salmonella* Typhi in Blantyre, Malawi—a resource limited, high burden setting. Between May 2021 and May 2022, *S*. Typhi was identified in both Moore swabs and grab samples collected across the city, providing evidence for active transmission and circulation of *S*. Typhi throughout the Blantyre population. We show that contaminated river networks can be used to conduct ES for *S*. Typhi in the absence of formal or informal sewage networks. At present, a standardised protocol for ES of *S*. Typhi in wastewaters and informal sewage network does not exist, therefore making direct comparison between studies challenging. However, various studies have used a range of sample collection and detection techniques to successfully identify *S*. Typhi in environmental samples [33,34].

A noteworthy advantage of our study is the relatively dense distribution of ES sites located throughout a mixed land use study settling, thus maximising surveillance coverage. Overall, rates of detection of *S*. Typhi using both grab samples and Moore swabs were relatively low. This is lower than other studies in different locations e.g. [35] in Kathmandu, although differing methods hinder comparability.

Whilst there was no evidence to indicate spatial clustering of *S*. Typhi positivity, we observed relatively higher rates of detection concentrated throughout the southwest of Blantyre, throughout the Mudi River basin draining the upstream Ndirande informal settlement. This densely populated informal settlement has minimal access to municipal services [36] where residents heavily rely on surface waters for domestic use [37] and has previously been identified as a typhoid high burden area [25,38]. Similar findings have been reported in urban river systems whereby elevated rates of *S*. Typhi detection were observed downstream of highly populated neighbourhoods [34].

A key challenge associated with sampling from an urban river network is the inconsistency with which the sampled water is contaminated with faecal matter: for example, there was evidence of human faecal contamination at all sites, but this was not consistent across time points. Measurement of the HF183 biomarker allowed us to calculate the prevalence of *S*. Typhi detection only among samples with evidence of human faecal contamination, which is likely to correlate more closely with incidence of infection in the community. Higher abundance of HF183 was associated with an increased probability of detection of *S*. Typhi. HF183 copy number could therefore be used to normalise the estimated abundance of *S*. Typhi based on qPCR, particularly in settings where sewage and human wastewater directly enter the river system.

We did not find an association between the prevalence or abundance of *S*. Typhi detection at ES sites and the incidence of typhoid fever by area of residence. Similarly, temporal peaks in detection did not align with peaks in typhoid fever incidence. However, it is noteworthy that the majority of *S*. Typhi positive ES samples, particularly for Moore swabs, were collected during the dry season between May and October 2021.While no significant link could be established between precipitation and *S*. Typhi detection, the detection of a fecally contaminated sample was slightly negatively correlated with rainfall, suggesting some dilution effects during periods of increased precipitation.

Overall, we found that rates of detection of *S*. Typhi were higher for Moore swabs compared to grab samples, though this difference was not statistically significant. The increased sensitivity might be because swabs may concentrate *S*. Typhi during a longer deployment period by filtering larger quantities of wastewater and/or because of the enrichment step during swab processing that is not performed for grab samples. The historical use of Moore swabs for ES, including Moore’s original use for typhoid, polio, norovirus, *Vibrio cholera*, *E*. *coli* [39–42] and recent use in SARS-CoV-2 surveillance [43], suggest that this low-cost and potentially highly sensitive surveillance method may have continued utility for multiple pathogens.

There are several limitations to our study. SARS-CoV-2 likely altered health seeking behaviours and the provision of blood culture to febrile cases, reducing recorded incidence of disease. Indeed, typhoid case numbers for the 2021 to 2022 period were lower than recent years. Similarly, the representativeness of crude incidence of blood culture confirmed cases of Typhoid fever is highly uncertain, given the low and variable diagnostic sensitivity (~59%, dependent on volume of blood drawn and recent antimicrobial use) [44] of blood culture tests, and low probability of an individual presenting at a clinic in Blantyre actually receiving a blood culture test due to limited capacity [12]. Our ability to draw comparisons between incidence and environmental detections is limited by these factors and by the potential for spatial heterogeneity in healthcare seeking behaviours, influenced, for example, by distance to the nearest healthcare facility [45]). Additionally, while it is possible that the typhoid burden was lower than usual due to changes in social mixing due to SARS-CoV-2, a study carried out in Blantyre in 2021 [35] found little reported social contact behavioural change. Geolocating cases with the same resolution as those of the ES sampling data was hampered by the absence of formalised addresses in many cases, meaning case locations could only be matched to ES sites based on the overlap of the administrative ward with the catchment area.

Here, we demonstrate the ability of ES to detect *S*. Typhi in an urban freshwater system. Whilst our data did not correlate with typhoid fever incidence, ES remains valuable for the longitudinal detection of *S*. Typhi in settings in which quality assured diagnostic microbiology facilities are lacking. Future work is needed in other settings to establish the link between the local incidence of typhoid fever and detection of *S*. Typhi in wastewater/sewage samples, which will capture shedding by both symptomatic and asymptomatic individuals. This could include comparison with active clinical surveillance systems and serological surveys.

Despite the absence of an association between *S*. Typhi in ES samples and reporting of typhoid fever cases, we found that ES could detect *S*. Typhi in an urban river system, highlighting continuous transmission. HF183 was inconsistently detected in ES samples and strongly associated with the detection of *S*. Typhi, highlighting the importance of ES site selection and sample type for the interpretation of ES data. We recommend that other settings seeking to establish the value of typhoid ES record this (or a similar) marker, avoiding the risk of river-water only samples biasing estimates, and follow consistent protocols for ES site selection, sample concentration and testing allowing comparison between settings, and where resources are limited, the selection of sites more likely to yield a fecally-contaminated sample. Anecdotally, bathing and clothes washing was observed close to some sampling sites. Future studies may benefit from formal collection of such data as a potential correlate of detection to be adjusted for in a similar way. While it was beyond the scope of this study to collect data pertaining to the use of and interaction with river water close to the sampling sites, given that typhoid has a fecal-oral transmission pathway, it may be valuable for future studies to assess how water at the sampling sites is used for washing clothes, drawing water, or cleaning kitchen utensils, especially as household use of river water for cooking and cleaning has been identified as a risk factor for Typhoid in Blantyre [25]. Overall, further studies of typhoid ES in these settings and comparison with active clinical surveillance and serological surveys will help to establish the value of typhoid ES for vaccine introduction and impact monitoring.

## Supporting information

S1 Table Positive and negative sample counts, by sample type. (DOCX)

S2 Table Parameter estimates for logistic regression model, with *S*. Typhi outcome ~ sample type + site specific random effects. (DOCX)

S3 Table Grab samples, univariate analysis. (DOCX)

S4 Table Moore swabs, univariate analysis. (DOCX)

S5 Table Moore swab multivariate analysis. (DOCX)

S6 Table Grab sample swab multivariate analysis. (DOCX)

S7 Table HF183 detection and covariates for grab samples. (DOCX)

S8 Table HF183 and covariates, Moore Swabs. (DOCX)

S9 Table Month by month results for *S*. Typhi detection, compared with recorded clinical case numbers. HF183 negative samples are excluded. (DOCX)

S1 Fig Monthly detection of HF183 in ES samples in Blantyre, Malawi between May 2021and May 2022. (TIF)

S2 Fig Heat maps showing geographic hotspots of *S*. Typhi detection in Blantyre. (PNG)

S3 Fig Estimated incidence of Typhoid fever within catchments, against proportions of samples which were positive for *S*. Typhi. (PNG)
