# Supplementary material for: Environmental surveillance for Salmonella Typhi in rivers and wastewater from an informal sewage network in Blantyre, Malawi
Source: PLoS Negl Trop Dis. 2024 Sep 27;18(9):e0012518. doi: 10.1371/journal.pntd.0012518 (PMC11463779; doi:10.1371/journal.pntd.0012518)
Supplement: S1 Table — (DOCX) [file pntd.0012518.s001.docx]

# S1 Table. Positive and negative sample counts, by sample type.

|  | Positive samples | Negative samples |
| --- | --- | --- |
| Moore Swab | 23 | 571 |
| Grab Sample | 11 | 522 |
