# Supplementary material for: Environmental surveillance for Salmonella Typhi in rivers and wastewater from an informal sewage network in Blantyre, Malawi
Source: PLoS Negl Trop Dis. 2024 Sep 27;18(9):e0012518. doi: 10.1371/journal.pntd.0012518 (PMC11463779; doi:10.1371/journal.pntd.0012518)
Supplement: S2 Table — (DOCX) [file pntd.0012518.s002.docx]

S2 Table. Parameter estimates for logistic regression model, with *S*. Typhi outcome ~ sample type + site specific random effects.

|  | Parameter Value | Sd | P value |
| --- | --- | --- | --- |
| Intercept | -3.95 | 0.399 | <2e-16 |
| Grab sample | -0.541 | 0.383 | 0.158 |
