# Supplementary material for: Environmental surveillance for Salmonella Typhi in rivers and wastewater from an informal sewage network in Blantyre, Malawi
Source: PLoS Negl Trop Dis. 2024 Sep 27;18(9):e0012518. doi: 10.1371/journal.pntd.0012518 (PMC11463779; doi:10.1371/journal.pntd.0012518)
Supplement: S3 Table — (DOCX) [file pntd.0012518.s003.docx]

S3 Table. Grab samples, univariate analysis.

| Covariate | Logistic regression parameter estimate | Standard deviation | 95% Confidence interval | Odds Ratio | 95% Confidence interval odds ratio | p- value |
| --- | --- | --- | --- | --- | --- | --- |
| Collection hours after 8am | Unable to fit model | | | | | |
| Temperature (C) | -0.03951 | 0.1128 | (-0.261,0.182) | 0.961 | (0.771,1.2) | 0.72614 |
| pH | 1.0176 | 0.86095 | (-0.67,2.71) | 2.77 | (0.512,15) | 0.23722 |
| Width of River | Unable to fit model | | | | | |
| Oxidation reduction potential (mV REDOX) | 0.0036227 | 0.0062469 | (-0.00862,0.0159) | 1 | (0.991,1.02) | 0.56197 |
| Natural log resitivity (K.Ohms.cm) | -0.070133 | 0.21079 | (-0.483,0.343) | 0.932 | (0.617,1.41) | 0.73935 |
| Salinity (PSU) | -0.41695 | 1.8454 | (-4.03,3.2) | 0.659 | (0.0177,24.5) | 0.82124 |
| HF183 presence | Unable to fit model: all samples positive for *S*. Typhi are positive for HF183. | | | | | |
| Natural log HF183 genome copies (gc/ul) | 0.28411 | 0.096495 | (0.095,0.473) | 1.33 | (1.1,1.61) | 0.0032365 |
| Catchment land use: residential low density (percentage) | 0.024299 | 0.017127 | (-0.00927,0.0579) | 1.02 | (0.991,1.06) | 0.15598 |
| Catchment land use: residential medium density (percentage) | -0.0045432 | 0.070324 | (-0.142,0.133) | 0.995 | (0.867,1.14) | 0.94849 |
| Catchment land use: residential high density traditional (percentage) | -0.11586 | 0.12666 | (-0.364,0.132) | 0.891 | (0.695,1.14) | 0.36033 |
| Catchment land use: residential high density permanent (percentage) | -0.00029077 | 0.028224 | (-0.0556,0.055) | 1 | (0.946,1.06) | 0.99178 |
| Catchment land use: residential high density informal (percentage) | -0.00062432 | 0.015389 | (-0.0308,0.0295) | 0.999 | (0.97,1.03) | 0.96764 |
| Catchment land use: commercial (percentage) | 0.10105 | 0.16998 | (-0.232,0.434) | 1.11 | (0.793,1.54) | 0.55218 |
| Catchment land use: industrial (percentage) | -0.0092181 | 0.044815 | (-0.0971,0.0786) | 0.991 | (0.908,1.08) | 0.83703 |
| Catchment land use: institutional (percentage) | 0.083829 | 0.06517 | (-0.0439,0.212) | 1.09 | (0.957,1.24) | 0.19834 |
| Catchment land use: utilities (percentage) | 0.44964 | 0.62678 | (-0.779,1.68) | 1.57 | (0.459,5.36) | 0.47314 |
| Catchment land use: residential low density (area, km sq) | -0.0015001 | 0.0026391 | (-0.00667,0.00367) | 0.999 | (0.993,1) | 0.56975 |
| Catchment land use: residential medium density (area, km sq) | -0.0148 | 0.021618 | (-0.0572,0.0276) | 0.985 | (0.944,1.03) | 0.49358 |
| Catchment land use: residential high density traditional (area, km sq) | 0.0012659 | 0.0049707 | (-0.00848,0.011) | 1 | (0.992,1.01) | 0.79898 |
| Catchment land use: residential high densityperm (area, km sq) | -0.0014563 | 0.0052165 | (-0.0117,0.00877) | 0.999 | (0.988,1.01) | 0.78012 |
| Catchment land use: residential high density informal (area, km sq) | -0.0017167 | 0.003355 | (-0.00829,0.00486) | 0.998 | (0.992,1) | 0.60888 |
| Catchment land use: commercial (area, km sq) | 0.0045621 | 0.017827 | (-0.0304,0.0395) | 1 | (0.97,1.04) | 0.79802 |
| Catchment land use: industrial (area, km sq) | 0.0013707 | 0.0033624 | (-0.00522,0.00796) | 1 | (0.995,1.01) | 0.68353 |
| Catchment land use: institutional (area, km sq) | 0.0021577 | 0.0059437 | (-0.00949,0.0138) | 1 | (0.991,1.01) | 0.71659 |
| Catchment land use: utilities (area, km sq) | -0.015009 | 0.04827 | (-0.11,0.0796) | 0.985 | (0.896,1.08) | 0.75584 |
| Pressure (Baro mb, scaled by taking away 880) | 0.011763 | 0.04781 | (-0.0819,0.105) | 1.01 | (0.921,1.11) | 0.80566 |
| Total dissolved solids (NTU) (scaled by 0.01) | -0.018359 | 0.1444 | (-0.301,0.265) | 0.982 | (0.74,1.3) | 0.89884 |
| Turbidity (mg/L) (scaled by 0.01) | -0.1113 | 0.29392 | (-0.687,0.465) | 0.895 | (0.503,1.59) | 0.70492 |
| Population in catchment (10 000s) | -0.054042 | 0.1025 | (-0.255,0.147) | 0.947 | (0.775,1.16) | 0.59801 |
| Speed of flow (Fast: slow+stagnant pooled reference category) | -0.25197 | 0.64794 | (-1.52,1.02) | 0.777 | (0.218,2.77) | 0.69736 |
| Depth of water (+50cm) | -0.39131 | 0.70848 | (-1.78,0.997) | 0.676 | (0.169,2.71) | 0.58072 |
| Type of site: Sewage site, river reference category. | 1.62 | 0.698 | (0.252,2.99) | 5.06 | (1.29,19.9) | 0.020271 |
| Total precipitation: day of sample | -0.09375 | 0.13281 | (-0.354;0.167) | 0.911 | (0.702;1.18) | 0.48028 |
| Total precipitation: day before sample | 0.009935 | 0.040982 | (-0.0704;0.0903) | 1.01 | (0.932;1.09) | 0.80844 |
| Total precipitation: 5-0 days before sample | -0.00096 | 0.01164 | (-0.0238;0.0219) | 0.999 | (0.977;1.02) | 0.93448 |
| Total precipitation: 6-1 days before sample | -0.00097 | 0.009587 | (-0.0198;0.0178) | 0.999 | (0.98;1.02) | 0.9192 |
