# Supplementary material for: Environmental surveillance for Salmonella Typhi in rivers and wastewater from an informal sewage network in Blantyre, Malawi
Source: PLoS Negl Trop Dis. 2024 Sep 27;18(9):e0012518. doi: 10.1371/journal.pntd.0012518 (PMC11463779; doi:10.1371/journal.pntd.0012518)
Supplement: S4 Table — (DOCX) [file pntd.0012518.s004.docx]

S4 Table. Moore swabs, univariate analysis.

| Covariate | | Logistic regression parameter estimate | Standard deviation | 95% Confidence interval | Odds Ratio | 95% Confidence interval odds ration | p-value |
| --- | --- | --- | --- | --- | --- | --- | --- |
| Collection hours after 8am | | 0.071141 | 0.12901 | (-0.182,0.324) | 1.07 | (0.834,1.38) | 0.58134 |
| Temperature (C) | | -0.07843 | 0.095779 | (-0.266,0.109) | 0.925 | (0.766,1.12) | 0.41287 |
| pH | | 0.76964 | 0.62243 | (-0.45,1.99) | 2.16 | (0.637,7.31) | 0.21626 |
| Width of River | <1m  >2m | 0.2958  -0.5520 | 1.1868  0.5140 | (-2.03,2.62)  (-1.56,0.455) | 1.34  0.576 | (0.131,13.8)  (0.210,1.58) | 0.803  0.283 |
| Oxidation reduction potential (mV REDOX) | | 0.010226 | 0.0056656 | (-0.000879,0.0213) | 1.01 | (0.999,1.02) | 0.071098 |
| Natural log resitivity (K.Ohms.cm) | | 0.12058 | 0.12929 | (-0.133,0.374) | 1.13 | (0.876,1.45) | 0.351 |
| Salinity (PSU) | | 0.40995 | 1.6829 | (-2.89,3.71) | 1.51 | (0.0557,40.8) | 0.80754 |
| HF183 presence | | 1.5286 | 0.5848 | (0.382,2.67) | 4.61 | (1.47,14.5) | 0.0089526 |
| Natural log HF183 genome copies (gc/ul) | | 0.44618 | 0.096365 | (0.257,0.635) | 1.56 | (1.29,1.89) | 0.0000036542 |
| Catchment land use: residential low density (percentage) | | 0.030836 | 0.01687 | (-0.00223,0.0639) | 1.03 | (0.998,1.07) | 0.067566 |
| Catchment land use: residential medium density (percentage) | | -0.0088372 | 0.070336 | (-0.147,0.129) | 0.991 | (0.864,1.14) | 0.90001 |
| Catchment land use: residential high density traditional (percentage) | | -0.011122 | 0.038306 | (-0.0862,0.064) | 0.989 | (0.917,1.07) | 0.77155 |
| Catchment land use: residential high density permanent (percentage) | | -0.0065845 | 0.02939 | (-0.0642,0.051) | 0.993 | (0.938,1.05) | 0.82273 |
| Catchment land use: residential high density informal (percentage) | | -0.029832 | 0.018344 | (-0.0658,0.00612) | 0.971 | (0.936,1.01) | 0.10389 |
| Catchment land use: commercial (percentage) | | 0.17712 | 0.17949 | (-0.175,0.529) | 1.19 | (0.84,1.7) | 0.32376 |
| Catchment land use: industrial (percentage) | | 0.0040033 | 0.03803 | (-0.0705,0.0785) | 1 | (0.932,1.08) | 0.91616 |
| Catchment land use: institutional (percentage) | | 0.12539 | 0.063445 | (0.00104,0.25) | 1.13 | (1,1.28) | 0.048118 |
| Catchment land use: utilities (percentage) | | 1.3353 | 0.51356 | (0.329,2.34) | 3.8 | (1.39,10.4) | 0.0093194 |
| Catchment land use: residential low density (area, km sq) | | 0.00078049 | 0.0018729 | (-0.00289,0.00445) | 1 | (0.997,1) | 0.67687 |
| Catchment land use: residential medium density (area, km sq) | | 0.0041427 | 0.0079536 | (-0.0114,0.0197) | 1 | (0.989,1.02) | 0.60247 |
| Catchment land use: residential high density traditional (area, km sq) | | -0.0014286 | 0.0063733 | (-0.0139,0.0111) | 0.999 | (0.986,1.01) | 0.82264 |
| Catchment land use: residential high densityperm (area, km sq) | | 0.0037288 | 0.0039656 | (-0.00404,0.0115) | 1 | (0.996,1.01) | 0.34707 |
| Catchment land use: residential high density informal (area, km sq) | | -0.0010407 | 0.0030435 | (-0.00701,0.00492) | 0.999 | (0.993,1) | 0.73239 |
| Catchment land use: commercial (area, km sq) | | 0.025318 | 0.017025 | (-0.00805,0.0587) | 1.03 | (0.992,1.06) | 0.13699 |
| Catchment land use: industrial (area, km sq) | | 0.002519 | 0.0033858 | (-0.00412,0.00916) | 1 | (0.996,1.01) | 0.45687 |
| Catchment land use: institutional (area, km sq) | | 0.0091271 | 0.0053531 | (-0.00136,0.0196) | 1.01 | (0.999,1.02) | 0.088192 |
| Catchment land use: utilities (area, km sq) | | 0.050742 | 0.035933 | (-0.0197,0.121) | 1.05 | (0.981,1.13) | 0.15791 |
| Pressure (Baro mb, scaled by taking away 880) | | 0.12944 | 0.078034 | (-0.0235,0.282) | 1.14 | (0.977,1.33) | 0.097165 |
| Total dissolved solids (NTU) (scaled by 0.01) | | -0.064266 | 0.11535 | (-0.29,0.162) | 0.938 | (0.748,1.18) | 0.57744 |
| Turbidity (mg/L) (scaled by 0.01) | | 0.10968 | 0.13306 | (-0.151,0.37) | 1.12 | (0.86,1.45) | 0.40976 |
| Population in catchment (10 000s) | | 0.03457 | 0.095835 | (-0.153,0.222) | 1.04 | (0.858,1.25) | 0.71831 |
| Speed of flow (Fast: slow+stagnant pooled reference category) | | 1.0355 | 0.47036 | (0.114,1.96) | 2.82 | (1.12,7.08) | 0.027693 |
| Depth of water (+50cm) | | 0.9008 | 0.4617 | (-0.00413,1.81) | 2.46 | (0.996,6.08) | 0.05105 |
| Type of site: Sewage site, river reference category. | | 1.82 | 0.808 | (0.239, 3.41) | 6.19 | (1.27,30.2) | 0.0241 |
| Total precipitation: day of sample | | -0.27769 | 0.26704 | (-0.801;0.246) | 0.758 | (0.449;1.28) | 0.2984 |
| Total precipitation: day before sample | | -0.18264 | 0.15428 | (-0.485;0.12) | 0.833 | (0.616;1.13) | 0.23648 |
| Total precipitation: 5-0 days before sample | | -0.01183 | 0.01718 | (-0.0455;0.0218) | 0.988 | (0.956;1.02) | 0.49103 |
| Total precipitation: 6-1 days before sample | | 0.010855 | 0.008927 | (-0.00664;0.0284) | 1.01 | (0.993;1.03) | 0.224 |
