# Supplementary material for: Environmental surveillance for Salmonella Typhi in rivers and wastewater from an informal sewage network in Blantyre, Malawi
Source: PLoS Negl Trop Dis. 2024 Sep 27;18(9):e0012518. doi: 10.1371/journal.pntd.0012518 (PMC11463779; doi:10.1371/journal.pntd.0012518)
Supplement: S5 Table — (DOCX) [file pntd.0012518.s005.docx]

S5 Table. Moore swab multivariate analysis.

| Covariate | Point estimate | Standard deviation | 95% Confidence interval | Odds Ratio | Odds ratio confidence interval | P-value |
| --- | --- | --- | --- | --- | --- | --- |
| Natural log HF183 genome copies per microlitre (gc/ul) | 0.413 | 0.0984 | (0.220,0.606) | 1.51 | (1.25,1.83) | 0.0000275 |
| Speed of flow: Fast. | 0.987 | 0.751 | (-0.485,2.46) | 2.68 | (0.615,11.7) | 0.189 |
| Depth of water: deep. | 0.362 | 0.647 | (-0.907,1.63) | 1.44 | (0.404,5.11) | 0.576 |
| Type of site: Sewage site, river reference category. | 0.112 | 0.655 | (-1.17,1.40) | 1.12 | (0.310,4.04) | 0.864 |
