# Supplementary material for: Environmental surveillance for Salmonella Typhi in rivers and wastewater from an informal sewage network in Blantyre, Malawi
Source: PLoS Negl Trop Dis. 2024 Sep 27;18(9):e0012518. doi: 10.1371/journal.pntd.0012518 (PMC11463779; doi:10.1371/journal.pntd.0012518)
Supplement: S6 Table — (DOCX) [file pntd.0012518.s006.docx]

# S6 Table. Grab sample swab multivariate analysis.

| Covariate | Point estimate | Standard deviation | 95% Confidence interval | Odds Ratio | Odds ratio confidence interval | P-value |
| --- | --- | --- | --- | --- | --- | --- |
| Natural log HF183 genome copies per microlitre (gc/ul) | 0.255 | 0.0942 | (0.0703,0.448) | 1.29 | (1.07,1.55) | 0.0068 |
| Type of site: Sewage site, river reference category. | 0.972 | 0.777 | (-0.552,2.49) | 2.64 | (0.576,12.1) | 0.211 |
