# Supplementary material for: Environmental surveillance for Salmonella Typhi in rivers and wastewater from an informal sewage network in Blantyre, Malawi
Source: PLoS Negl Trop Dis. 2024 Sep 27;18(9):e0012518. doi: 10.1371/journal.pntd.0012518 (PMC11463779; doi:10.1371/journal.pntd.0012518)
Supplement: S7 Table — (DOCX) [file pntd.0012518.s007.docx]

S7 Table. HF183 detection and covariates for grab samples.

| Covariate | | Logistic regression parameter estimate | Standard deviation | | 95% Confidence interval | | | Odds Ratio | 95% Confidence interval odds ration | | p-value |
| --- | --- | --- | --- | --- | --- | --- | --- | --- | --- | --- | --- |
| Collection hours after 8am | | -0.040513 | 0.056645 | | (-0.152,0.0705) | | | 0.96 | (0.859,1.07) | | 0.47448 |
| Temperature (C) | | 0.030027 | 0.039261 | | (-0.0469,0.107) | | | 1.03 | (0.954,1.11) | | 0.44439 |
| pH | | 0.28022 | 0.25499 | | (-0.22,0.78) | | | 1.32 | (0.803,2.18) | | 0.2718 |
| Width.of.River | <1m  >2m | 1.3015  -0.0782 | | 0.79553  0.216 | | (-0.258, 2.86)  (-0.502,0.346) | 3.67  0.925 | | (0.773,17.5)  (0.605,1.41) | 0.10183  0.717 | |
| Oxidation reduction potential (mV REDOX) | | 0.0057777 | 0.0026642 | | (0.000556,0.011) | | | 1.01 | (1,1.01) | | 0.030111 |
| Natural log resitivity (K.Ohms.cm) | | 0.12369 | 0.082904 | | (-0.0388,0.286) | | | 1.13 | (0.962,1.33) | | 0.1357 |
| Salinity (PSU) | | -0.16787 | 0.75005 | | (-1.64,1.3) | | | 0.845 | (0.194,3.68) | | 0.8229 |
| Catchment land use: residential low density (percentage) | | -0.00055788 | 0.0077989 | | (-0.0158,0.0147) | | | 0.999 | (0.984,1.01) | | 0.94297 |
| Catchment land use: residential medium density (percentage) | | -0.0065686 | 0.022131 | | (-0.0499,0.0368) | | | 0.993 | (0.951,1.04) | | 0.76661 |
| Catchment land use: residential high density traditional (percentage) | | -0.012932 | 0.010416 | | (-0.0333,0.00748) | | | 0.987 | (0.967,1.01) | | 0.21438 |
| Catchment land use: residential high density permanent (percentage) | | 0.0067843 | 0.0094996 | | (-0.0118,0.0254) | | | 1.01 | (0.988,1.03) | | 0.47512 |
| Catchment land use: residential high density informal (percentage) | | -0.0080558 | 0.0049465 | | (-0.0178,0.00164) | | | 0.992 | (0.982,1) | | 0.1034 |
| Catchment land use: commercial (percentage) | | 0.18541 | 0.067385 | | (0.0533,0.317) | | | 1.2 | (1.05,1.37) | | 0.0059315 |
| Catchment land use: industrial (percentage) | | 0.011712 | 0.013545 | | (-0.0148,0.0383) | | | 1.01 | (0.985,1.04) | | 0.3872 |
| Catchment land use: institutional (percentage) | | 0.095678 | 0.027695 | | (0.0414,0.15) | | | 1.1 | (1.04,1.16) | | 0.00055094 |
| Catchment land use: utilities (percentage) | | 0.66879 | 0.24791 | | (0.183,1.15) | | | 1.95 | (1.2,3.17) | | 0.0069809 |
| Catchment land use: residential low density (area, km sq) | | 1.211e-05 | 0.00065937 | | (-0.00128,0.0013) | | | 1 | (0.999,1) | | 0.98535 |
| Catchment land use: residential medium density (area, km sq) | | 0.0029315 | 0.0031121 | | (-0.00317,0.00903) | | | 1 | (0.997,1.01) | | 0.3462 |
| Catchment land use: residential high density traditional (area, km sq) | | 3.9831e-05 | 0.0019346 | | (-0.00375,0.00383) | | | 1 | (0.996,1) | | 0.98357 |
| Catchment land use: residential high density permanent (area, km sq) | | 0.0032124 | 0.001597 | | (8.23e-05,0.00634) | | | 1 | (1,1.01) | | 0.044267 |
| Catchment land use: residential high density informal (area, km sq) | | -0.00099877 | 0.00094509 | | (-0.00285,0.000854) | | | 0.999 | (0.997,1) | | 0.2906 |
| Catchment land use: commercial (area, km sq) | | 0.013434 | 0.006285 | | (0.00112,0.0258) | | | 1.01 | (1,1.03) | | 0.032556 |
| Catchment land use: industrial (area, km sq) | | 0.00039743 | 0.0012733 | | (-0.0021,0.00289) | | | 1 | (0.998,1) | | 0.75495 |
| Catchment land use: institutional (area, km sq) | | 0.005756 | 0.0021981 | | (0.00145,0.0101) | | | 1.01 | (1,1.01) | | 0.0088275 |
| Catchment land use: utilities (area, km sq) | | 0.033023 | 0.014569 | | (0.00447,0.0616) | | | 1.03 | (1,1.06) | | 0.023413 |
| Pressure (Baro mb, scaled by taking away 880) | | -0.0083575 | 0.016234 | | (-0.0402,0.0235) | | | 0.992 | (0.961,1.02) | | 0.60668 |
| Total dissolved solids (NTU) (scaled by 0.01) | | -0.033404 | 0.052459 | | (-0.136,0.0694) | | | 0.967 | (0.873,1.07) | | 0.52428 |
| Turbidity (mg/L) (scaled by 0.01) | | 0.087796 | 0.085625 | | (-0.08,0.256) | | | 1.09 | (0.923,1.29) | | 0.3052 |
| Population in catchment (10 000s) | | 0.030333 | 0.033114 | | (-0.0346,0.0952) | | | 1.03 | (0.966,1.1) | | 0.35966 |
| Speed of flow (Fast: slow+stagnant pooled reference category) | | 0.48592 | 0.19936 | | (0.0952,0.877) | | | 1.63 | (1.1,2.4) | | 0.01479 |
| Depth of Water (+50cm) | | 0.221 | 0.21116 | | (-0.193,0.635) | | | 1.25 | (0.825,1.89) | | 0.29529 |
| Type of site: Sewage site, river reference category. | | 1.03 | 0.521 | | (0.00515,2.05) | | | 2.80 | (1.01,7.78) | | 0.0489 |
| Total precipitation: day of sample | | -0.0443 | 0.016452 | | (-0.0765;-0.0121) | | | 0.957 | (0.926;0.988) | | 0.007092 |
| Total precipitation: day before sample | | 0.009308 | 0.013977 | | (-0.0181;0.0367) | | | 1.01 | (0.982;1.04) | | 0.50543 |
| Total precipitation: 5-0 days before sample | | 0.004618 | 0.003573 | | (-0.00239;0.0116) | | | 1 | (0.998;1.01) | | 0.19619 |
| Total precipitation: 6-1 days before sample | | 0.004969 | 0.002937 | | (-0.000788;0.0107) | | | 1 | (0.999;1.01) | | 0.090704 |
