# Supplementary material for: Environmental surveillance for Salmonella Typhi in rivers and wastewater from an informal sewage network in Blantyre, Malawi
Source: PLoS Negl Trop Dis. 2024 Sep 27;18(9):e0012518. doi: 10.1371/journal.pntd.0012518 (PMC11463779; doi:10.1371/journal.pntd.0012518)
Supplement: S8 Table — (DOCX) [file pntd.0012518.s008.docx]

S8 Table. HF183 and covariates, Moore Swabs.

| Covariate | | Logistic regression parameter estimate | Standard deviation | 95% Confidence interval | Odds Ratio | 95% Confidence interval odds ration | p-value |
| --- | --- | --- | --- | --- | --- | --- | --- |
| Collection hours after 8am | | 0.073171 | 0.057242 | (-0.039,0.185) | 1.08 | (0.962,1.2) | 0.20115 |
| Temperature (C) | | 0.0094994 | 0.039374 | (-0.0677,0.0867) | 1.01 | (0.935,1.09) | 0.80936 |
| pH | | -0.29394 | 0.2555 | (-0.795,0.207) | 0.745 | (0.452,1.23) | 0.24995 |
| Width.of.River | <1m  >2m | 0.26467  -0.195 | 0.67034  0.219 | (-1.05,1.58)  (-0.624,0.233) | 1.3  0.823 | (0.35,4.85)  (0.536,1.26) | 0.69297  0.371 |
| Oxidation reduction potential (mV REDOX) | | -0.0031933 | 0.0023613 | (-0.00782,0.00143) | 0.997 | (0.992,1) | 0.17627 |
| Natural log resitivity (K.Ohms.cm) | | 0.0067397 | 0.083394 | (-0.157,0.17) | 1.01 | (0.855,1.19) | 0.93559 |
| Salinity (PSU) | | 0.088817 | 0.68397 | (-1.25,1.43) | 1.09 | (0.286,4.18) | 0.89668 |
| Catchment land use: residential low density (percentage) | | 0.0063433 | 0.011253 | (-0.0157,0.0284) | 1.01 | (0.984,1.03) | 0.57297 |
| Catchment land use: residential medium density (percentage) | | 0.048034 | 0.031852 | (-0.0144,0.11) | 1.05 | (0.986,1.12) | 0.13154 |
| Catchment land use: residential high density traditional (percentage) | | -0.022262 | 0.016634 | (-0.0549,0.0103) | 0.978 | (0.947,1.01) | 0.1808 |
| Catchment land use: residential high density permanent (percentage) | | 0.024042 | 0.012622 | (-0.000697,0.0488) | 1.02 | (0.999,1.05) | 0.056808 |
| Catchment land use: residential high density informal (percentage) | | -0.012242 | 0.0070946 | (-0.0261,0.00166) | 0.988 | (0.974,1) | 0.084423 |
| Catchment land use: commercial (percentage) | | 0.13162 | 0.093039 | (-0.0507,0.314) | 1.14 | (0.951,1.37) | 0.15716 |
| Catchment land use: industrial (percentage) | | -0.012595 | 0.018743 | (-0.0493,0.0241) | 0.987 | (0.952,1.02) | 0.50158 |
| Catchment land use: institutional (percentage) | | 0.15086 | 0.035891 | (0.0805,0.221) | 1.16 | (1.08,1.25) | 2.6323e-05 |
| Catchment land use: utilities (percentage) | | 1.5215 | 0.31571 | (0.903,2.14) | 4.58 | (2.47,8.5) | 1.4419e-06 |
| Catchment land use: residential low density (area, km sq) | | 2.369e-05 | 0.0010068 | (-0.00195,0.002) | 1 | (0.998,1) | 0.98123 |
| Catchment land use: residential medium density (area, km sq) | | 0.013216 | 0.004522 | (0.00435,0.0221) | 1.01 | (1,1.02) | 0.0034704 |
| Catchment land use: residential high density traditional (area, km sq) | | 0.00042997 | 0.0028928 | (-0.00524,0.0061) | 1 | (0.995,1.01) | 0.88184 |
| Catchment land use: residential high density permanent (area, km sq) | | 0.0081527 | 0.002173 | (0.00389,0.0124) | 1.01 | (1,1.01) | 0.00017558 |
| Catchment land use: residential high density informal (area, km sq) | | -0.003427 | 0.001444 | (-0.00626,-0.000597) | 0.997 | (0.994,0.999) | 0.017634 |
| Catchment land use: commercial (area, km sq) | | 0.02305 | 0.0081202 | (0.00713,0.039) | 1.02 | (1.01,1.04) | 0.0045308 |
| Catchment land use: industrial (area, km sq) | | -0.00059114 | 0.001856 | (-0.00423,0.00305) | 0.999 | (0.996,1) | 0.75011 |
| Catchment land use: institutional (area, km sq) | | 0.012317 | 0.0028943 | (0.00664,0.018) | 1.01 | (1.01,1.02) | 2.0846e-05 |
| Catchment land use: utilities (area, km sq) | | 0.083418 | 0.018697 | (0.0468,0.12) | 1.09 | (1.05,1.13) | 8.1367e-06 |
| Pressure (Baro mb, scaled by taking away 880) | | 0.0024662 | 0.020184 | (-0.0371,0.042) | 1 | (0.964,1.04) | 0.90275 |
| Total dissolved solids (NTU) (scaled by 0.01) | | -0.0062781 | 0.05132 | (-0.107,0.0943) | 0.994 | (0.899,1.1) | 0.90264 |
| Turbidity (mg/L) (scaled by 0.01) | | -0.029609 | 0.078064 | (-0.183,0.123) | 0.971 | (0.833,1.13) | 0.70447 |
| Population in catchment (10 000s) | | 0.04236 | 0.048119 | (-0.052,0.137) | 1.04 | (0.949,1.15) | 0.37869 |
| Speed of flow (Fast: slow+stagnant pooled reference category) | | 0.26463 | 0.19863 | (-0.125,0.654) | 1.3 | (0.883,1.92) | 0.18277 |
| Depth of Water (+50cm) | | 0.10365 | 0.22423 | (-0.336,0.543) | 1.11 | (0.715,1.72) | 0.6439 |
| Type of site: Sewage site, river reference category. | | 1.75 | 0.613 | (0.543, 2.95) | 5.73 | (1.72, 19.1) | 0.00443 |
| Total precipitation: day of sample | | -0.03879 | 0.017625 | (-0.0733;-0.00424) | 0.962 | (0.929;0.996) | 2.7757e-02 |
| Total precipitation: day before sample | | 0.01587 | 0.016251 | (-0.016;0.0477) | 1.02 | (0.984;1.05) | 3.2880e-01 |
| Total precipitation: 5-0 days before sample | | 0.000302 | 0.00549 | (-0.0105;0.0111) | 1 | (0.99;1.01) | 9.5616e-01 |
| Total precipitation: 6-1 days before sample | | 0.004559 | 0.004902 | (-0.00505;0.0142) | 1 | (0.995;1.01) | 3.5237e-01 |
|  | |  |  |  |  |  |  |
