# Supplementary material for: Environmental surveillance for Salmonella Typhi in rivers and wastewater from an informal sewage network in Blantyre, Malawi
Source: PLoS Negl Trop Dis. 2024 Sep 27;18(9):e0012518. doi: 10.1371/journal.pntd.0012518 (PMC11463779; doi:10.1371/journal.pntd.0012518)
Supplement: S9 Table — (DOCX) [file pntd.0012518.s009.docx]

S9 Table. Month by month results for *S*. Typhi detection, compared with recorded clinical case numbers. HF183 negative samples are excluded.

| Month | Moore Swab  Excluding HF183 negative samples | | Grab Sample  Excluding HF183 negative samples | | Clinical cases  Total (Hospital only) |
| --- | --- | --- | --- | --- | --- |
| May 2021 | 4/27 | 14.8% | 2/32 | 6.25% | 7 (5) |
| June | 1/30 | 3.33% | 1/18 | 5.56% | 13 (11) |
| July | 6/42 | 14.33% | 1/22 | 4.55% | 4 (2) |
|  |  |  |  |  |  |
| August | 5/33 | 15.2% | 1/27 | 3.70% | 7 (3) |
| September | 2/19 | 10.5% | 1/24 | 4.17% | 13 (10) |
| October | 0/20 | 0 | 1/23 | 4.35% | 9 (8) |
| November | 0/24 | 0 | 1/24 | 4.17% | 12 (4) |
| December | 0/25 | 0 | 0/18 | 0 | 8 (7) |
| January 2022 | 0/2 | 0 | 1/25 | 4.00% | 8 (7) |
| February | 0/13 | 0 | 1/38 | 2.63% | 14 (12) |
| March | 0/19 | 0 | 1/38 | 2.63% | 17 (13) |
| April | 1/9 | 11.1% | 0/27 | 0 | 10 (8) |
| May | 0/14 | 0 | 0/26 | 0 | No data |
