# Supplementary figures and images for: Environmental surveillance for Salmonella Typhi in rivers and wastewater from an informal sewage network in Blantyre, Malawi
Source: PLoS Negl Trop Dis. 2024 Sep 27;18(9):e0012518. doi: 10.1371/journal.pntd.0012518 (PMC11463779; doi:10.1371/journal.pntd.0012518)

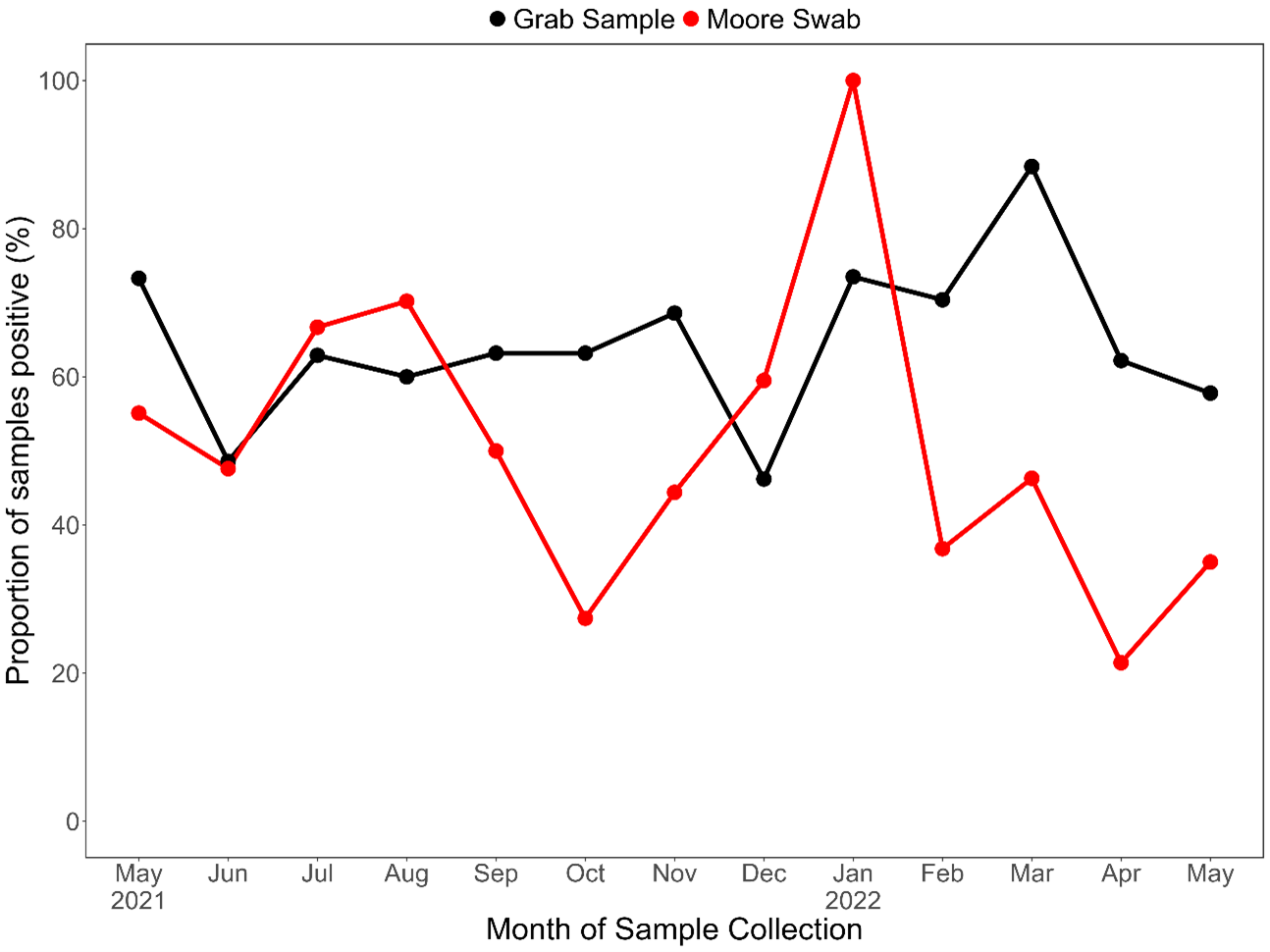

Supplement: S1 Fig — (TIF) [file pntd.0012518.s010.tif]

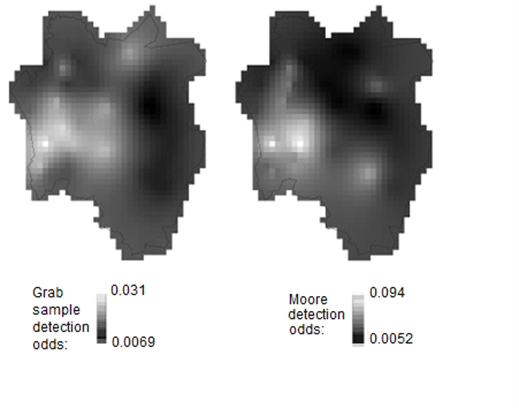

Supplement: S2 Fig — (PNG) [file pntd.0012518.s011.png]

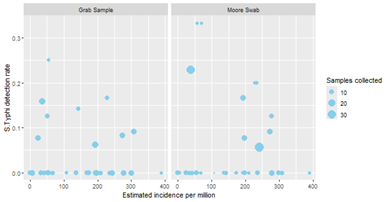

Supplement: S3 Fig — (PNG) [file pntd.0012518.s012.png]
